# Enhancing Text Message Support With Media Literacy and Financial Incentives for Vaping Cessation in Young Adults: Protocol for a Pilot Randomized Controlled Trial

**DOI:** 10.2196/60527

**Published:** 2025-02-21

**Authors:** Tzeyu Michaud, Troy Puga, Rex Archer, Elijah Theye, Cleo Zagurski, Paul Estabrooks, Hongying Daisy Dai

**Affiliations:** 1 Department of Health Promotion College of Public Health University of Nebraska Medical Center Omaha, NE United States; 2 Center for Reducing Health Disparities College of Public Health University of Nebraska Medical Center Omaha, NE United States; 3 Department of Orthopaedic Surgery Medical City Denton Denton, TX United States; 4 College of Osteopathic Medicine Kansas City University Kansas city, MO United States; 5 Office of the Dean College of Public Health University of Nebraska Medical Center Omaha, NE United States; 6 Health Administration and Policy Program Creighton University Omaha, NE United States; 7 Department of Health and Kinesiology College of Health University of Utah Salt Lake City, UT United States; 8 Department of Biostatistics College of Public Health University of Nebraska Medical Center Omaha, NE United States

**Keywords:** contingency management, e-cigarettes, social support, youth, electronic health record, opt-in, recruitment, tobacco marketing, cessation, peer support, young adult, feasibility, public health

## Abstract

**Background:**

The persistent high prevalence of e-cigarette use among young adults remains a significant public health concern, with limited evidence and guidance on effective vaping cessation programs targeting this population.

**Objective:**

This study aims to outline the study design and protocol of a pilot randomized controlled trial aimed at investigating feasibility and assessing whether media literacy education or financial incentives enhance the effectiveness of evidence-based text message support in promoting vaping abstinence among young adult e-cigarette users.

**Methods:**

The pilot study uses a 4-arm (1:1:1:1) randomized controlled trial design to assess the potential impact of different combinations of media literacy education, financial incentives, and text message support on vaping abstinence over a 3-month period. The first month serves as a preparatory phase for quitting, followed by 2 months focused on abstinence. A total of 80 individuals, aged 19-29 years, who have used e-cigarettes within the past 30 days, have internet access, and express interest in quitting vaping within the next 30 days, will be enrolled. Eligible individuals will be randomized into one of the four study groups: (1) Text Message, (2) Media Literacy, (3) Financial Incentive, and (4) Combined. All participants, regardless of group assignment, will receive text message support. Participants will be followed for 12 weeks, with abstinence status assessed at week 12, as well as during remote check-ins at weeks 6, 8, and 10. Feasibility measures include recruitment rate, reach, engagement, and retention. Other outcomes of interest include self-reported 7-day abstinence and changes in nicotine dependence and media literacy scores. Exit interviews will be conducted with those who complete the study to explore facilitators of and barriers to participation and engagement in vaping cessation, which will inform future program refinement and uptake.

**Results:**

Recruitment for the study commenced in December 2023 and concluded in August 2024. A total of 40 participants were randomized into these groups: 9 for Text Message, 11 for Media Literacy, 10 for Financial Incentive, and 10 for the Combined group. The final assessment was completed in November 2024, and analyses are currently ongoing.

**Conclusions:**

The findings from this trial could provide valuable insights into the design and uptake of vaping cessation strategies among the young adult population.

**Trial Registration:**

ClinicalTrials.gov NCT05586308; https://clinicaltrials.gov/study/NCT05586308

**International Registered Report Identifier (IRRID):**

DERR1-10.2196/60527

## Introduction

E-cigarettes have emerged as the most commonly used tobacco product among young individuals [1,2], rapidly becoming a public health concern in the United States, particularly among youth and young adults [3]. Data from the 2021 National Center for Health Statistics reported that e-cigarette use (ie, vaping) was highest among young adults aged 18-24 years, with 11% (approximately 3.4 million people) using e-cigarettes, compared with an overall adult rate of 4.5% [4]. The 2023 National Center for Health Statistics reported an increase in overall adult e-cigarette use to 6.5% [5]. Similarly, another study using the 2021 Behavioral Risk Factor Surveillance System Survey observed a high prevalence of e-cigarette use among US adults (6.9%, 95% CI 6.7%-7.1%), particularly among young adults aged 18-24 years (18%) [6]. The accessibility of vaping products has significantly increased, largely driven by marketing efforts featuring appealing flavors targeted at this population [7]. Moreover, the influence of social media and influencers has contributed to the widespread prevalence of vaping [8,9]. Given the persistently high rates of e-cigarette use, especially among young adults, it is critical to explore the feasibility and effectiveness of targeted cessation programs for this group.

Although marketed as a safer alternative to combustible cigarettes [1,7], evidence suggests that vaping still poses health risks [10,11]. Vaping has been associated with increases in both blood pressure and heart rates [12]. Nicotine, a toxic and highly addictive substance found in e-cigarettes, contributes to these risks, with concentrations varying widely depending on the type of devices, their power, and the brands and models. Notably, nicotine levels in e-cigarettes have increased from 1.7% in 2017 to 5.0% in 2022 [13]. However, critical questions regarding both the short-term and long-term health effects of e-cigarette use remain unanswered [14-16].

Vaping cessation efforts among young adults often yield poor results, with many experiencing failed attempts [17,18]. Data indicate that 53.4% of current young e-cigarette users express an intention to quit, and 67.4% report attempts to do so [19], yet successful cessation rates remain low [20]. Despite a strong desire to quit, many struggle to stop vaping. Evidence-based behavioral vaping cessation programs are scarce compared with pharmacological interventions, such as nicotine replacement therapies [21-23] or vape concentration tapering [24]. Moreover, the implementation and reach of these behavioral programs remain a challenge, particularly among youth and young adult populations [25].

Three recent studies have reported on the effectiveness of behavioral vaping cessation programs using text message support or financial incentives. For example, Raiff et al [26] conducted a pre-post intervention study with 8 college students, finding that all participants quit vaping during the 2-week intervention period, using a remotely delivered program with financial incentives to reinforce abstinence. However, in a feasibility trial involving 27 young adults, Palmer et al [27] found no significant difference in vaping abstinence between the contingency management and monitoring control groups, despite the positive feedback about the intervention program. Another study examined a vaping cessation text message program called This is Quitting [28], which provides social support for quitting. In a randomized clinical trial (RCT) involving 2588 young adults aged 18-24 years, participants in the This is Quitting group were 1.39 times more likely to quit vaping compared with those in the control group [29]. The urgent need for evidence-based behavioral vaping cessation initiatives among this population is critical to addressing this public health concern.

Vaping often appeals to younger populations through marketing campaigns that highlight various flavors and glamorize the practice [7]. Companies also use manipulative strategies like social media influencers and product placement, particularly targeting communities with lower levels of vaping-related media literacy, who tend to be more susceptible to vaping [30]. Although limited evidence exists on the direct impact of media literacy programs on vaping cessation, integrating such programs into vaping cessation initiatives may help promote abstinence by (1) promoting positive behavioral norms (educating people about the true risks of vaping can foster a social environment where quitting is seen as important and achievable [31]); (2) encouraging peer advocacy and mentoring (by understanding marketing tactics and gaining skills to debunk misinformation, individuals can become more effective in supporting their peer to quit vaping [32]); and (3) enhancing self-efficacy against vaping triggers (media literacy can help individuals recognize and avoid triggers that encourage vaping, such as targeted advertising or social media content that normalizes the behaviors [33]). In addition, although media literacy programs are primarily designed for youth for educational and prevention purposes, young adults may also benefit from the content due to the close age proximity between these groups.

Contingency management is a well-established behavioral intervention in which individuals receive reinforcement, such as financial incentives, contingent upon predetermined outcomes [25]. Financial incentives have been shown to be effective in promoting healthy behaviors [34] and improving the reach and engagement of evidence-based programs as part of an implementation strategy [35]. Numerous studies have demonstrated the success of financial incentives in encouraging behavior changes [36,37]. Rooted in health promotion economic theory, financial incentives enhance the perceived benefits of abstaining from unhealthy behaviors, thereby bolstering motivation for change [36].

Peer influence plays an important role in vaping behaviors, with family and friend use cited as key reasons for vaping initiation [38]. In addition, vaping for “entertainment” purposes is prevalent among younger populations [39]. Peer support strategies have been shown to be effective [40,41], especially in promoting cigarette smoking cessation in youth and young adults [42,43]. Platforms like SMS text messaging and social media have shown promise as peer support tools [44-46]. Specifically, peer support delivered through text message (eg, This is Quitting) has demonstrated effectiveness in vaping cessation across multiple studies [29,47,48], capitalizing on the widespread use of these communication methods among young adults.

The objective of this paper is to outline the design and methodology of a pilot RCT investigating feasibility and assessing whether media literacy education or financial incentives enhance the effectiveness of evidence-based text message support in promoting vaping abstinence among young adult e-cigarette users.

## Methods

### Study Design

This pilot study uses a 4-arm RCT design to assess whether media literacy education or financial incentives can enhance vaping abstinence when combined with text message support over a 3-month trial period. The first month involves a preparatory phase for quitting, followed by 2 months focused on maintaining abstinence from vaping. Eligible participants will be randomly assigned to one of four groups in a 1:1:1:1 ratio: (1) Text Message (text message support), (2) Media Literacy (text message support and media literacy education), (3) Financial Incentive (text message support and financial incentives), and (4) Combined (text message support, media literacy education, and financial incentives).

All 4 groups will receive evidence-based text message support (ie, This is Quitting). In addition, to develop and assess the process of conducting biospecimen collection (ie, urine) and analysis, a subsample of 20 participants (5 participants from each study group) will be contacted in order of enrollment until the target is met at baseline. These participants will be asked to provide urine samples both at baseline and at the conclusion of the study. The samples will be shipped to the Division of Laboratory Sciences at the Centers for Disease Control and Prevention for biomarker analyses to evaluate exposures to tobacco-related toxicants from e-cigarette use.

The trial protocol has been reported in accordance with the SPIRIT (Standard Protocol Items Recommendations for Interventional Trials) 2013 checklist [49] (Multimedia Appendix 1).

### Participants and Eligibility Criteria

Eligible participants are individuals who: (1) are aged 19-29 years (the minimum age is set at 19 to align with the legal age of majority in Nebraska); (2) have used e-cigarettes within the past 30 days, consistent with previous behavioral vaping cessation trials targeting youth and young adults [29,50]; (3) have access to the internet; and (4) are interested in quitting vaping within the next 30 days. Individuals who self-report as currently pregnant or planning to become pregnant within the next 3 months, or who are currently enrolled in other behavioral or medical vaping cessation programs, will be excluded.

### Sample Size Justification

As this pilot RCT aims to assess the feasibility of a future large-scale study, a sample size of 80 (n=20 in each arm) is considered sufficient, following the flat rule of thumb for pilot trials with continuous outcomes of interest (eg, feasibility outcomes), which typically recommend group sizes ranging from 12 to 35 participants [51]. This sample size also takes into account available resources such as time and budget [52].

### Recruitment and Sample Selection

We will recruit a total of 80 participants using a population health management approach [53,54], a comprehensive strategy that involves identifying subpopulations of individuals from an existing pool who would benefit from a given evidence-based intervention. This approach includes examining the characteristics of these populations using electronic health record (EHR) data. The potential participant pool will be limited to patients who have opted in to be contacted for research studies in the EHR system at Nebraska Medicine.

Specifically, we will collaborate with EHR Data Access Core at the University of Nebraska Medical Center to first generate a list of potentially eligible participants, filtering for age and using smart text data to determine the vaping or smoking status, as young adults are more likely to engage in multiple tobacco products at the same time [55]. A study invitation email, containing a link to an eligibility screening, will then be sent to individuals on this potential participant list by the study team. In this email, interested individuals will be directed to complete web-based screening questions via Research Electronic Data Capture (REDCap; Vanderbilt University) to assess their eligibility. Eligible participants will then be contacted by research staff members on days 1, 3, 7, 14, and 21 (if no response) after the completion of screening to schedule an in-person baseline visit.

If the recruitment goal of 80 participants is not met after exhausting the initial participant list, the research team will directly contact potential participants via phone calls. In addition, we will implement a social media recruitment strategy by posting study advertisements on platforms such as Facebook (Meta), Instagram (Meta), or X (formerly Twitter). We recognize that there may be differences between participants recruited from various sources (EHR vs social media) and will account for these differences in the reporting of trial results and the planning of future large-scale studies.

### Interventions

#### Text Message Support

Grounded in social cognitive theory and designed as a compassionate, nonjudgmental companion, the text message program “This is Quitting” is an evidence-based, cost-free, and personalized initiative by the Truth Initiative, aimed at helping young individuals quit vaping. Most of the support messages are contributed by other users, reinforcing perceived social norms and providing communal encouragement for quitting. A recent RCT involving 2588 young adults aged 18-24 years found that participants receiving text message support were significantly more likely to achieve vaping abstinence compared with the control group (odds ratio [OR] 1.39; 95% CI 1.15-1.68; *P*<.001) at the 7-month follow-up [29]. Participants will retain full access to the text message program even after completing the study.

Participants in this study will receive 1 message per day for the week leading up to their quit date and for 8 weeks following it.

#### Media Literacy

Media Education for Sensible Evaluation and Nurturing Substance-free Experiences (MediaSense) [56] is an evidence-informed antivaping media literacy education program designed to prevent and reduce vaping among adolescents and young adults. Developed by our research team, the program draws on the social influence framework and incorporates guidance from the Centers for Disease Control and Prevention and Food and Drug Administration tobacco and vaping prevention guides, as well as an extensive review of evidence-based prevention programs and identified vaping risk factors. The MediaSense program consists of 9 web-based modules covering a range of topics, including (1) understanding what e-cigarettes are and how they work; (2) exploring the health impact of e-cigarettes; (3) understanding the role of marketing and advertisement in promoting e-cigarettes; (4) dispelling myths and revealing facts about e-cigarette marketing; and (5) learning how to deconstruct vaping advertisements. These e-learning modules aim to transform participants’ knowledge, attitudes, and beliefs regarding e-cigarette use while emphasizing vaping-related media literacy and developing skills to critically deconstruct e-cigarette advertisements.

In our previous study that examined the effect of MediaSense on vaping prevention among middle and high school students (n=384), we observed significant results. Specifically, vaping media literacy scores improved, with mean scores increasing from 2.3 (SD 2.3) to 3.4 (SD 2.4) (*P*<.001). In addition, there was a decrease in the perception of vaping harm (adjusted OR 1.6 95% CI 1.1-1.22) and a reduction in vaping susceptibility (adjusted OR 0.7, 95% CI 0.5-1.0; *P*=.04) [56], indicating a reduced likelihood of students considering vaping.

In this study, participants assigned to Media Literacy or Combined will complete these self-paced modules during the 1-month quitting preparatory phase and will be required to take a short quiz at the end of each module. These quizzes confirm module completion and assess participants’ understanding of the education materials. Each module will take approximately 10 minutes to complete.

#### Financial Incentives

**Figure 1:**
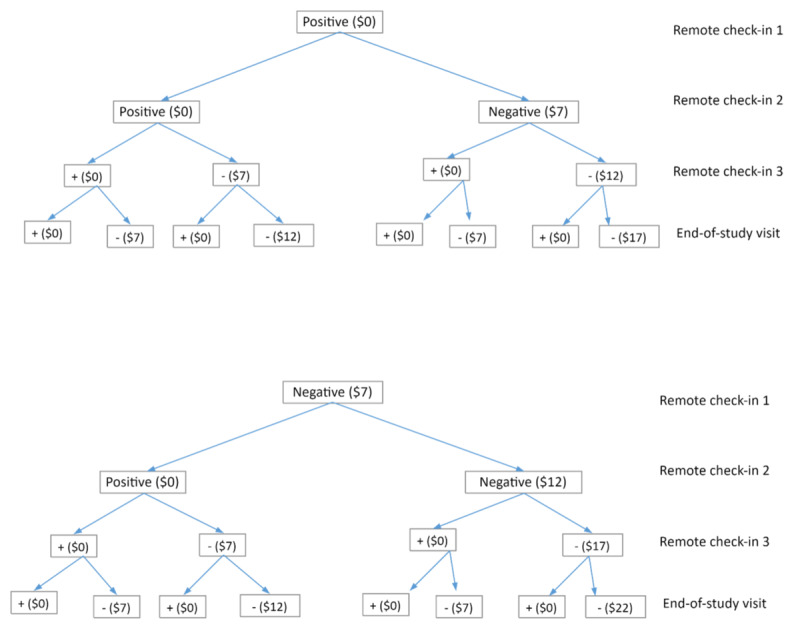
Financial reward decision flow. Values were negative (-) or positive (+).

Participants will receive US $3 for each saliva sample submission, regardless of testing results, at week 6, week 8, week 10, and week 12 during the 2-month abstinence phase. In addition, they will earn escalating bonuses for each negative sample. The bonus will start at US $7 and increase by US $5 for each subsequent negative sample, that will be, US $7 for week 6, US $12 for week 8, US $17 for week 10, and US $22 for week 12. However, a reset contingency will be applied, meaning the bonus amount will revert to the initial US $7 if a sample is missing or tests positive for cotinine (refer to Figure 1).

Participants can earn up to $70 in total upon completing all 4 saliva sample submissions. They will be informed of their earnings after completing each scheduled task (ie, immediate reward) and will receive all payments as a 1-time disbursement at the conclusion of the study. The rationale behind the timing and amount of the incentives is to minimize the risk of inhibiting intrinsic motivation [57], as modest incentives (up to US $70) are less likely to undermine intrinsic motivation compared with the larger rewards typically offered in smoking cessation programs (eg, US $1185) [58].

### Study Timeline and Data Collection

**Figure 2:**
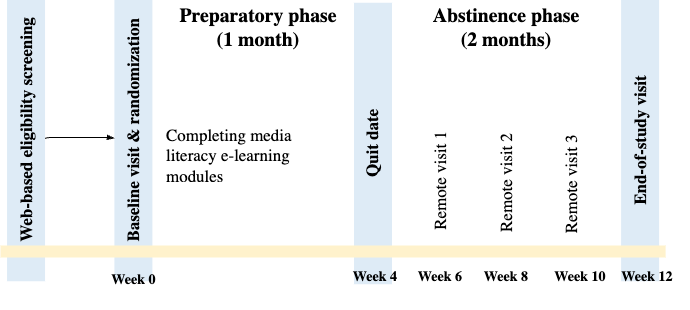
Study assessment timepoint.

The study will span a duration of 3 months, consisting of a 1-month quitting preparatory phase (weeks 1-4) and a 2-month abstinence phase (weeks 5-12; Figure 2). Throughout the quitting preparatory phase, participants assigned to the Media Literacy or Combined groups will be directed to complete the web-based media literacy education modules by their quit date, Meanwhile, participants in the Text Message or Financial Incentive groups will be encouraged to prepare for quitting but not to quit before the specified quite date.

#### Baseline Visit

This assessment will include a demonstration and facilitation of the saliva test, which participants will perform themselves at baseline and final study visits, as well as during remote check-ins. Participants will also sign a precommitment pledge (a psychological strategy to help individuals stick with their goals) and complete the baseline survey.

Following these activities, research staff members will conduct randomization using predetermined blocks of 4 and 8, with a computer-generated algorithm, to assign participants to one of the study groups. Participants will also be asked to specify a quit date (no later than 1 month after the baseline visit, which serves as the quitting preparatory phase), register for the text message program, and schedule the 3 remote check-ins for saliva testing.

#### Remote Check-Ins

During the abstinence phase, participants will engage in a total of 3 video calls, scheduled for week 6, week 8, and week 10. During each live video call, participants will receive instructions on how to complete the saliva sample collection on their own and submit the results. Specifically, they will be directed to open a new test kit and record their participant ID, visit number, date, and time on the kit. Participants will then perform the saliva test, allowing approximately 10 minutes for processing. In addition to verbally providing the test results (positive or negative) to the research staff members, participants will be required to take a picture of the results, including their participant ID, visit number, date, and time, and send it to the research staff members before concluding the call. Each video call will last no longer than 20 minutes. The primary purpose of these remote check-ins is to maintain participants’ engagement and monitor their abstinence progress until the final study visit at week 12.

#### End-of-Study Visit

At the end-of-study visit (week 12), which may be conducted either remotely or in person, participants will provide the results of saliva testing and complete the end-of-study survey. Participants who provided a urine sample at the study baseline will be asked to attend the final visit in person for urine sample collection.

#### Exit Interview

For quality improvement and future modification and adoption of vaping cessation programs, we will conduct exit interviews with participants (n=8-12; 2-3 participants from each group) who have completed the study via video call. The interview guide will be developed based on the Pragmatic Robust Implementation and Sustainability Framework [59] to identify the barriers and facilitators of participating in a vaping cessation program and achieving abstinence goals.

### Measures

#### Feasibility Outcome

As this is a pilot trial, our primary objective is to assess the feasibility of implementation. Specifically, we will use study records to measure the following: (1) recruitment rate, defined as the proportion of eligible individuals who consent to participate; (2) reach, assessed by the representativeness of sociodemographic characteristics among screened and enrolled individuals; (3) engagement, measured by the proportion of participants completing remote check-ins, and the proportion of participants in the Media Literacy or Combined groups who complete the media literacy e-learning modules; (4) retention, evaluated by the number of participants who withdraw from study, and the completeness of the end-of-study assessment; and (5) the number and proportion of participant providing a urine sample from each group at baseline and the end of study.

#### Cessation Outcome

The primary cessation outcome for this study is biochemically verified vaping abstinence, determined through the iScreen saliva cotinine test conducted at the end-of-study assessment. A negative cotinine result will be indicated by a cutoff of <30 ng/mL [60].

#### Additional Outcome

Additional outcomes of interest for this study include: (1) self-reported 7-day abstinence, determined by the question in the end-of-study survey: “Have you used any e-cigarettes, even a puff or pinch, in the last 7 days?” [61]; (2) changes in nicotine dependence, measured by the Penn State E-Cigarette Dependence Index at baseline and end-of-study; and (3) changes in media literacy, assessed using the Vaping Media Literacy Scale [30] at baseline and end-of-study.

### Data Analysis Plan

#### Quantitative Analysis

Descriptive statistics will be used to summarize participants’ sociodemographic characteristics, e-cigarette use history, exposure to tobacco marketing and its influence, social support, and self-efficacy at baseline. Bivariate analyses, using either the Chi-squared test for categorical variables or the ANOVA test for continuous variables, will be conducted to identify differences in sociodemographic characteristics and other vaping-related variables at baseline across the study groups.

We will provide a descriptive summary of feasibility outcomes, including the rate of recruitment, reach, engagement, and retention. In addition, we will assume that all covariate data are missing at random, and this assumption will be tested by comparing the missing data patterns with sociodemographic characteristics in the bivariate analyses.

Chi-squared tests will be used to determine differences in proportions of participants with negative saliva test results at the end of the study as well as each remote check-in. Missing end-of-study abstinence outcomes will be coded as vaping (not abstinent) [27]. Similarly, differences in the proportion of participants who self-reported 7-day abstinence across study groups will be evaluated using the Chi-squared test, while the ANOVA test will be used to assess changes in nicotine dependence index and media literacy scores.

Nonparametric methods (eg, Fisher exact test when the sample size is less than 5 in a category or the Kruskal-Wallis test) will be applied when appropriate. All quantitative analyses will be conducted in SAS (version 9.4; SAS Institute Inc) with a 2-tailed significance level <.05 if applicable.

#### Qualitative Analysis

Using a hybrid deductive and inductive qualitative approach, we will analyze data obtained from semistructured interviews to explore the facilitators and barriers to participation and engagement in vaping cessation programs. All interviews will be recorded and transcribed verbatim. Subsequently, 2 trained research staff members will independently code the transcripts into reduced meaning units (sentences or phases with singular meaning). Intercoder reliability will be assessed using Cohen κ, with a threshold of ≥.80. All qualitative data analyses will be conducted using NVivo 14 (Lumivero) or Microsoft Excel.

### Ethical Considerations

The study protocol was approved by the institutional review board (0596-22-EP) at the University of Nebraska Medical Center and was registered on ClinicalTrials.gov (NCT05586308).

#### Informed Consent

Approximately 10 days before the baseline visit, eligible individuals scheduled for the in-person visit will receive an email containing an initial packet with the informed consent form, a letter with instructions for their first visit, and contact information for any questions. During the baseline visit, trained research staff members or study investigators will verbally review the informed consent with participants, covering the data collection procedure and plans for protecting participants and their privacy (ie, data deidentification), address any questions, and obtain written informed consent before proceeding with the baseline assessment.

For participants who opt to provide a urine sample during the baseline visit, verbal consent will be obtained by the research staff members before the sample collection. Participants will be guided through the recommended procedures for urine sample collection according to best practices.

#### Compensation

All study participants will receive a US $25 gift card each for completing baseline and end-of-study visits. Participants who opt to provide a urine sample will receive an additional US $10 at both baseline and end-of-study visits. As a result, participants in the Text Message or Media Literacy groups can earn up to US $70 in total compensation, while those in the Financial Incentive or Combined groups may earn up to US $140, including additional financial rewards contingent on the submission of 4 saliva samples during remote check-ins or end-of-study visit.

## Results

Recruitment for the study commenced in December 2023 and concluded in August 2024. A total of 40 participants were randomized into the following groups: 9 for Text Message, 11 for Media Literacy, 10 for Financial Incentive, and 10 for the Combined group. The final assessment was completed in November 2024, and analyses are currently ongoing.

## Discussion

### Principal Findings

This study is one of the few to assess the feasibility and potential effects of different vaping cessation program combinations in helping young adults quit vaping. We anticipate that the addition of either media literacy or financial incentives will lead to better preliminary abstinence outcomes (ie, more participants quit vaping) by the end of the study, compared with text message support alone.

Unlike other behavioral trials for vaping cessation, which often use a “doing nothing” approach (ie, assessment-only) for the control condition [28,50], we aim to enhance vaping abstinence outcomes by examining the potential additive effects of various interventions on top of an evidence-based program. This approach accounts for participants’ expectation of receiving some levels of interventions when taking part in research studies [28].

There are currently few published studies on behavioral interventions for young adult vaping cessation [26,27,29]. As of the time of writing, 9 other clinical trial protocols are actively recruiting participants to evaluate behavioral vaping cessation interventions among youth (n=3), young adults (n=4), or a mixed youth and young adult population (n=1). These trials have target sample sizes ranging from 30 to 1715 participants, as listed in their registration records on ClinicalTrials.gov. Given the persistently high prevalence of e-cigarette use among young adults, it is crucial to identify effective cessation programs and the supplementary components that may enhance their success. The study findings will be disseminated through peer-reviewed manuscript publications and presentations at national conferences, as well as shared with relevant stakeholders and communities.

### Limitations

This study has several limitations that warrant acknowledgment. First, the exclusion of individuals without access to internet services may inadvertently overlook those most in need of cessation assistance, particularly those of low socioeconomic status or residing in remote areas. However, studies have indicated that approximately 95% of American adults have internet access and 90% own smartphones [62], suggesting that the benefits of using technology-assisted behavioral interventions likely outweigh the drawbacks of this exclusion criterion. Second, our use of a commercially available saliva cotinine test kit, which provides binary positive or negative results, to verify abstinence status may not fully capture the extent to which cotinine levels have fluctuated as a result of cessation efforts. Nonetheless, the saliva test offers cost-efficiency compared with urine testing and may serve as a useful monitoring tool for follow-up assessments during the intervention period. Third, although we apply a population health management approach to identify potential participants from an EHR database, it is likely that we are capturing only individuals who receive health care within a specific system rather than from a broader regional population. In addition, due to institutional policy, we are limited to including only those individuals who have opted in for research participation, which may introduce selection bias, as these individuals may be less representative of the broader target population [63]. Furthermore, concerns exist regarding the reliability of using vaping or smoking status as a filter to identify potential participants from the EHR database. To address this, we have incorporated an additional step in the recruitment process, requiring potential participants to complete a screening survey to confirm their eligibility before scheduling the baseline assessment. Finally, while our study targets young adults aged 19-29 years, the text message program (This is Quitting) is tailored for individuals aged 18-24 years. We believe that individuals within the broader age range can still benefit from the support program. To accommodate this, participants aged 25-29 years in the trial will be instructed to adjust their age when registering for the text message program during the baseline visit.

### Conclusion

In summary, the findings from this trial will aid in refining the study design, particularly regarding vaping program components, and in planning program implementation. In addition, the results may help identify or develop strategies to enhance participant reach and program uptake among young adult populations.

## Appendix

Multimedia Appendix 1 The SPIRIT checklist.

## Abbreviations

EHR: electronic health record
MediaSense: Media Education for Sensible Evaluation and Nurturing Substance-free Experiences
OR: odds ratio
RCT: randomized clinical trial
SPIRIT: Standard Protocol Items: Recommendations for Interventional Trials

## Data Availability

Data sharing is not applicable to this article, as no datasets were generated or analyzed during this study due to the trial being ongoing.
